# Establishment and application of multiplex droplet digital polymerase chain reaction assay for bovine enterovirus, bovine coronavirus, and bovine rotavirus

**DOI:** 10.3389/fvets.2023.1157900

**Published:** 2023-09-12

**Authors:** Junzhen Chen, Dan Li, Yafang Xu, Zeyu Li, Siqi Ma, Xinyi Liu, Yuanyuan Yuan, Chengyuan Zhang, Qiang Fu, Huijun Shi

**Affiliations:** ^1^College of Veterinary Medicine, Xinjiang Agricultural University, Ürümqi, China; ^2^Tecon Biology Co., Ltd., Ürümqi, China

**Keywords:** bovine enterovirus, bovine coronavirus, bovine rotavirus, droplet digital PCR, real-time quantitative PCR

## Abstract

Bovine enterovirus (BEV), bovine coronavirus (BCoV), and bovine rotavirus (BRV) are still the major worldwide concerns in the health care of cattle, causing serious economic losses in the livestock industry. It is urgent to establish specific and sensitive methods to detect viruses for the early control of diseases. Droplet digital PCR (ddPCR) has been proposed to effectively detect viral particles, and it does not involve Ct values or standard curves. In this study, we designed specific primers and probes, based on conserved regions of viral genomes, to optimize protocols for a dual ddPCR assay for detecting BCoV and BRV and a multiplex ddPCR assay for BEV, BCoV, and BRV. Sensitivity assays revealed that the lower limit of detection for qPCR was 1,000 copies/μL and for ddPCR for BEV, BCoV, and BRV, 2.7 copies/μL, 1 copy/μL and 2.4 copies/μL, respectively. Studying 82 samples collected from diarrheal calves on a farm, our dual ddPCR method detected BCoV, BRV, and co-infection at rates of 18.29%, 14.63%, and 6.1%, respectively. In contrast, conventional qPCR methods detected BCoV, BRV, and co-infection at rates of 10.98%, 12.2%, and 3.66%, respectively. On the other hand, studying 68 samples from another farm, qPCR detected BCoV, BRV, BEV, and co-infection of BCoV and BEV at rates of 14.49%, 1.45%, 5.80%, and 1.45%, respectively. Our multiplex ddPCR method detected BCoV, BRV, BEV, co-infection of BCoV and BEV, and co-infection of BRV and BEV. at rates of 14.49%, 2.9%, 8.7%, 2.9%, and 1.45%, respectively. Studying 93 samples from another farm, qPCR detected BCoV, BRV, BEV, and co-infection of BCoV and BEV was detected at rates of 5.38%, 1.08%, 18.28%, and 1.08%, respectively. Co-infection of BCoV, BRV, BEV, BCoV, and BEV, and co-infection of BRV and BEV, were detected by multiplex ddPCR methods at rates of 5.38%, 2.15%, 20.45%, 1.08%, and 1.08%, respectively. These results indicated that our optimized dual and multiplex ddPCR methods were more effective than conventional qPCR assays to detect these viral infections.

## 1. Introduction

Bovine enterovirus (BEV) and bovine coronavirus (BCoV) are single-stranded positive-strand RNA viruses (1, 2). BEV infection in cattle causes diarrhea, bloody stools, respiratory distress, decreased milk production, and infertility (3, 4). BCoV causes diarrhea in calves and winter diarrhea and respiratory disease in adult cattle (5). Bovine rotavirus (BRV) is a double-stranded RNA virus (6). BRV infects calves to produce watery diarrhea, depression, and loss of appetite, leading to a morbidity rate of 90% to 100% and a mortality rate of 50% (7, 8). BCoV and BRV are able to infect an animal. BCoV and BRV are also able to infect humans (2, 9). As a serious concern, BEV, BCoV, and BRV have spread into most areas of China (10–12). Thus, it is urgent to establish a reliable, specific, and quantitative method to effectively detect and differentiate these viruses for early control of viral infections and timely prevention of their spreading.

In 1985, Mullis and Faloona (13) reported the first generation of the polymerase chain reaction (PCR) method. In 1992, Higuchi et al. (14) proposed the second generation of real-time quantitative PCR (qPCR), which became an improved method to detect pathogens quantitatively, specifically, sensitively, and reproducibly for clinical diagnosis of animal/human diseases and food quality concerns. However, the qPCR method involves the construction of standard curves and Ct values (15). In 1999, Vogelstein and Kinzler (16) proposed the third generation digital PCR (dPCR) capable of detecting low quantities of multiple targets effectively without relying on the standard curve and Ct values (17). Recently, Nyaruaba’s group (18) reported the multiplex ddPCR assay for the detection of SARS-CoV-2. This new method helps develop an advanced diagnostic assay for SARS-CoV-2 with reduced false positive and false negative results. It also helps laboratory research to maximize the number of targets to be detected within a small volume of sample (19). However, the multiplex ddPCR technique has not been applied to the simultaneous detection of BEV, BCoV, and BRV. Thus, in this research, we pursued the development of simple, rapid, specific, and sensitive hybrid ddPCR assays to detect and differentiate infections by BEV, BCoV, and BRV.

## 2. Materials and methods

### 2.1. Clinical tissue samples and main reagents

Samples were obtained from 82 diarrheic calves from a cattle farm in Ürümqi, Xinjiang, China; 68 anal subsamples were obtained from diarrheic cattle from a cattle farm in Bole, Xinjiang, China; and 93 anal formulae were collected from cattle in cattle farms in Ürümqi County, Xinjiang, China.

TRIzol was purchased from Invitrogeng; 2× Taq PCR Mix, Common Agarose Gel DNA Recovery Kit (DP209), DH5α receptor cells, Rapid Plasmid Small Extraction Kit, and SuperReal Fluorescence Quantitative Premix Reagent (TaqMan) were purchased from Tiangen Biochemical Technology Co., Ltd. (Beijing, China); RT EasyTM II Reverse Transcription Kit was purchased from FIC Biotechnology Co., Ltd. (Chengdu, China); T-Vector pMD19 vector kit was purchased from TaKaRa; 2× ddPCR SuperMix for Probes was purchased from Bio-Rad.

### 2.2. Construction of BEV, BCoV, and BRV standard plasmids

According to the GenBank database, combined with the published sequences of BEV-E, BCoV N^pro^, and the BRV-VP6 gene, the sequences were sent to Sangon Bioengineering (Shanghai) Co., Ltd. BEV, BCoV, and BRV PCR were cloned into pMD18-T, pGEM-T Easy Vector, and pUC18 vectors (purchased from ADDGENG), sequenced for validation, and mapped (Supplementary Figure S1). The specific PCR reaction system is shown in Supplementary Table S1. Reaction conditions were 95°C, 5 min; 95°C, 30 s; annealing, 30 s; 72°C, 1 min; 34 cycles; and 72°C, 10 min.

### 2.3. Design of BEV, BCoV, and BRV ddPCR primers and probes

The correctly sequenced pMD18-T-BEV-E, pGEM-T-BCoV-N, and pUC18-BRV-VP6 positive recombinant plasmids were used as templates according to the GenBank database. Primer Premier 5.0 (Primer Canada Inc.) software was used to design specific primers and probes for the conserved regions of BEV E (GenBank No. MN607031.1), BCoV N^pro^ (GenBank No. LC494174.1), and BRV-VP6 (GenBank No. AB573082.1) genes for qPCR and ddPCR reactions (Supplementary Table S2). The probe sequence was sent to Suzhou GENEWIZ Biotechnology Co., Ltd. for synthesis.

### 2.4. Establishment of the TaqMan real-time quantitative PCR method

According to the SuperReal Pre Mix (TaqMan) kit instructions, plasmid standards of BEV, BCoV, and BRV at a concentration of 1 × 10^6^ copies/μL were selected as templates, and other conditions were controlled constants. qPCR reactions were performed at the upstream and downstream primer concentrations, selected as 200, 300, 400, 500, 600, 700, 800, and 900 nM concentration gradients, to screen for the best concentration of primers to use. The qPCR reactions were performed when the final concentrations of probes were set to 150, 200, 250, 300, 350, 400, and 450 nM, respectively, to determine the optimal probe concentrations for BEV, BCoV, and BRV. The initial concentrations of 1 × 10^10^ copies/μL of BEV, BCoV, and BRV plasmid standards were diluted in a 10-fold gradient (1 × 10^9^–1 × 10^2^ copies/μL), qPCR reactions were performed, standard curves were constructed, and sensitivity, specificity, and reproducibility tests were performed. The PCR program was 94°C for 3 min, 94°C for 7 s, 60°C for 30 s, and 40 cycles.

### 2.5. Sensitivity detection

BEV, BCoV, and BRV were diluted in a 10-fold gradient (1 × 10^9^–1 × 10^2^ copies/μL), and a negative control was set up; each concentration was repeated three times. qPCR reactions were performed using the optimized reaction conditions to determine the sensitivity of the method.

### 2.6. Specificity detection

The specificity of the qPCR method was assessed by the established qPCR method for the detection of positive plasmids of BEV, BCoV, and BRV, respectively, with a single standard plasmid as the positive control, water as the negative control, and the rest of the standard plasmids as the detection group for the testing.

### 2.7. Repeatability detection

The optimized method was used to perform qPCR reactions using 1 × 10^7^ copies/μL, 1 × 10^6^ copies/μL and 1 × 10^5^ copies/μL of plasmid standards as template concentrations, which were repeated three times. At intervals, three replicate experiments were performed using the above conditions to detect the batch-to-batch variation in the method. The coefficient of variation (CV) of Ct values in intra- and inter-batch replicate experiments was calculated using the formula CV = standard deviation/mean, and the CV was used to respond to reproducibility.

### 2.8. Establishment of BEV, BCoV, and BRV single ddPCR detection method

The ddPCR reaction was performed using the QX200 ddPCR system of the Bio-Rad Company, program was 94°C for 10 min, 94°C for 30 s, 58°C for 30 s, 72°C for 30 s, 40 cycles, 98°C for 10 min, and 4°C for storage. The reaction system was configured according to the ddPCR SuperMix for Probes kit (Bio-Rad) to ensure complete mixing and no air bubbles. The droplets were placed into the reader for low signal readings. Sensitivity, specificity, and repeatability experiments were carried out.

### 2.9. Establishment of BCoV and BRV dual ddPCR detection reactions

According to the ddPCR workflow and method, primers and probes for BCoV and BRV were used. The FAM channel detected the target BCoV, and the HEX channel detected the target BRV. The duplex ddPCR reaction system was: 2× ddPCR SuperMix for Probes 10 μL, two pairs of upper, downstream primer concentrations were 450 nM each, probe concentrations were each 250 nM, the template was 2 μL, ddH_2_O was supplemented to 20 μL, and the reaction conditions were the same as above.

### 2.10. Optimization of multiplex ddPCR reaction conditions for BEV, BCoV, and BRV

The annealing temperature was set to 58°C, the primers were all 500 nM, and four groups of different probe concentrations were set. The conditions of the reactions were optimized. The specific probe sequences and concentrations are shown in Supplementary Table S3.

### 2.11. RNA extraction and reverse transcription

Stool samples were added to three times the volume of sterilized phosphate-buffered saline (PBS), vortexed and mixed, and centrifuged at 10,000 g for 10 min at 4°C. The supernatant was collected, penicillin-streptomycin liquid was added and left for 4 h in a refrigerator at 4°C, and the virus treatment solution was obtained by filtration using a 0.22 μm filter. The total RNA of the samples was extracted using TRIzol (Invitrogen), the concentration of total RNA was determined, and the total RNA was reverse transcribed to cDNA using a reverse transcription kit.

### 2.12. Clinical sample detection

The 82 fresh samples collected from diarrheal calves from the cattle farm in Ürümqi were added to the sterilized PBS, vortexed, and mixed. The extracted RNA was reverse transcribed into cDNA. These samples were then tested using the constructed qPCR and duplex ddPCR methods. The 68 fresh samples collected from diarrheal calves from the cattle farm in Bole were treated with sterilized PBS, vortexed, and mixed, and RNA was extracted and reverse transcribed into cDNA. These samples were then examined using established qPCR and multiplex ddPCR methods.

## 3. Results

### 3.1. Establishment of BEV, BCoV, and BRV qPCR methods

**Figure 1:**
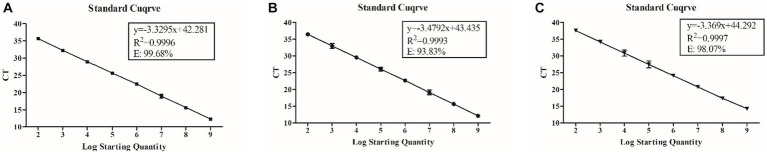
Regression equation, correlation coefficient (*R*^2^), and amplification efficiency of standard curves for BEV, BCoV, and BRV qPCR assays. **(A)** Standard curve of BEV with a primer concentration of 200 nM and a probe concentration of 400 nM. **(B)** Standard curve of BCoV with a primer concentration of 300 nM and a probe concentration of 150 nM. **(C)** Standard curve of BRV with a primer concentration of 300 nM and a probe concentration of 500 nM. Horizontal coordinate: number of copies **(A,B)** or logarithm of the number of copies **(C)**; vertical coordinate: cycle threshold **(A,B)**; equation: standard curve equation; *R*^2^: correlation coefficient; E: amplification efficiency.

Plasmid standards of BEV, BCoV, and BRV were used as templates, and gradient concentrations of upstream and downstream primers were set for qPCR reactions. The optimal primer concentrations for BEV, BCoV, and BRV were found to be 200 nM, 300 nM, and 300 nM, respectively, according to the analysis based on the principle of producing the smallest Ct value and the most economical dosage (Supplementary Table S4). The optimal probe concentrations were 400 nM for BEV, 150 nM for BCoV, and 500 nM for BRV (Supplementary Table S5). The standard curves were constructed with the above conditions and showed regular variations between each concentration range and the Ct values, all with excellent linearity. The regression equation of the standard curve for BEV was *y* = −0.3295*x* + 42.281; the correlation coefficient (*R*^2^) of the standard curve was 0.9996, and the amplification efficiency was 99.68% (Figure 1A). The regression equation of the standard curve for BCoV was *y* = −3.4792*x* + 43.435, *R*^2^ = 0.9993, and the amplification efficiency was 93.83% (Figure 1B). The regression equation of the standard curve of BRV was *y* = −3.369*x* + 44.292, *R*^2^ = 0.9997, and the amplification efficiency was 98.07% (Figure 1C).

### 3.2. Sensitivity detection

**Figure 2:**
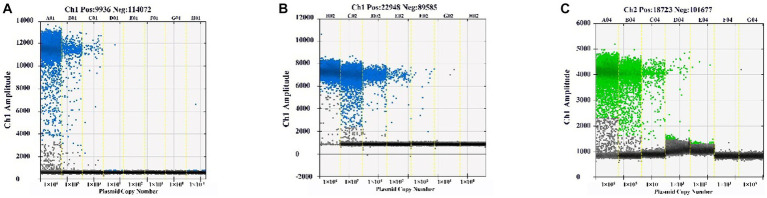
Sensitivity of the ddPCR assay. The standards of BEV, BCoV, and BRV were diluted to 1 × 10^9^–1 × 10^2^ copies/μL in a 10-fold gradient and assayed by the constructed ddPCR method. **(A)** Sensitivity assay of the BEV ddPCR method. **(B)** Sensitivity assay of the BCoV ddPCR method. **(C)** Sensitivity assay of the BRV ddPCR method. Horizontal coordinate: plasmid copy number; vertical coordinate: fluorescence amplitude. Ch1 amplitude: FAM, respectively.

The plasmid standards of BEV, BCoV, and BRV were diluted to 1 × 10^9^–1 × 10^2^ copies/μL using the 10-fold dilution method, and qPCR and ddPCR sensitivity experiments were performed. qPCR assay results showed that the Ct values of BEV, BCoV, and BRV plasmid concentrations were less than 35 at 1,000 copies/μL, so the lower limit of detection of the method was 1,000 copies/μL (Supplementary Table S6). The ddPCR assay results showed that the lowest detection concentrations of the established ddPCR assays for BEV, BCoV, and BRV were 2.7 copies/μL (Figure 2A), 1 copy/μL (Figure 2B), and 2.4 copies/μL (Figure 2C), respectively. The sensitivity of the established ddPCR assays for BEV, BCoV, and BRV was shown to be 370.37, 1,000, and 416.67 times higher than that of the corresponding qPCR assays, indicating that ddPCR is significantly more sensitive than qPCR and is able to detect lower levels of virus and reduce false negatives.

### 3.3. Specificity detection

**Figure 3:**
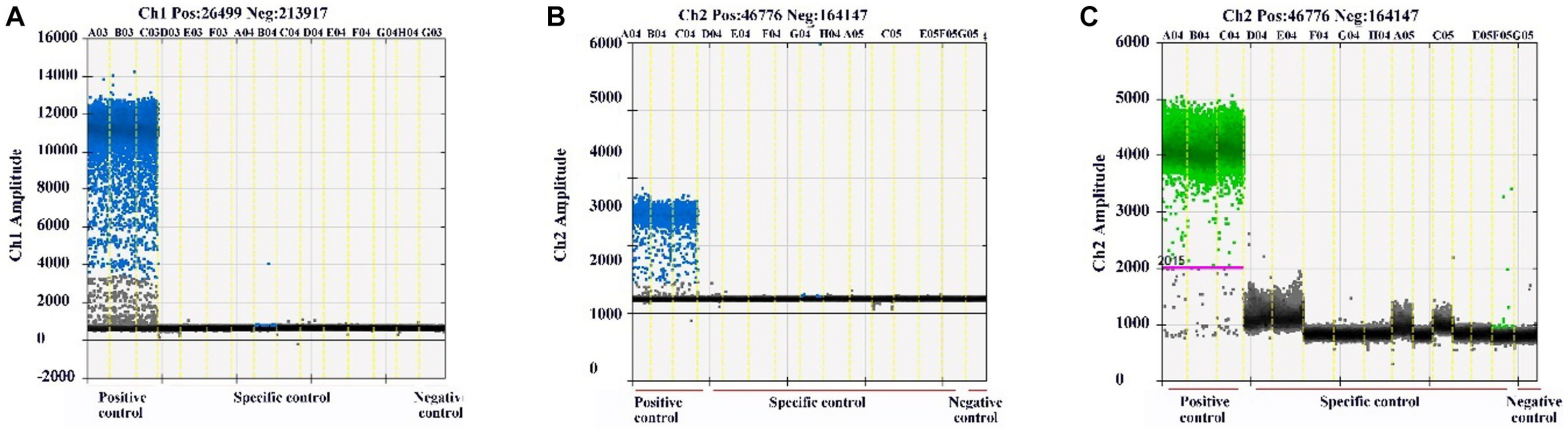
Specificity detection results of ddPCR assays. Specificity detection of BEV, BCoV, BRV, and H_2_O using established ddPCR assays. **(A)** Specificity detection of BEV using ddPCR. **(B)** Specificity detection of BCoV using ddPCR. **(C)** Specificity detection for BRV ddPCR. Horizontal coordinate: set experimental group; vertical coordinate: fluorescence amplitude. Ch1 amplitude: FAM, respectively.

The specificity of the qPCR and ddPCR assays was evaluated with the Plasmids of BEV, BCoV, BRV, BVDV, and H_2_O as templates. qPCR results showed that only the corresponding virus positive control had an amplification curve, while the other viruses did not (Supplementary Figures S2A–C). ddPCR results showed that only the corresponding virus produced positive droplets, while the other viruses did not (Figures 3A–C), indicating that both assays had good specificity.

### 3.4. Repeatability detection

Intra- and inter-batch replicate tests of the qPCR assay were performed on standard plasmids from 1 × 10^7^ to 1 × 10^5^ copies/μL. The results showed that the CVs of intra- and inter-batch replicate tests for BEV, BCoV, and BRV (Supplementary Table S7) were less than 3%. BEV intra- and inter-batch reproducibility tests were performed by diluting BEV plasmid standards to 1.52 × 10^5^ and 1.52 × 10^6^ copies/μL. Intra- and inter-batch CVs were less than 1.3%, which was 0.5 times higher than that of qPCR. BCoV reproducibility tests were performed by diluting BCoV plasmid standards to 3.95 × 10^5^ and 3.95 × 10^6^ copies/μL. The intra- and inter-batch CVs were less than 5.1%, which was 2.13 times higher than that of qPCR. BRV reproducibility tests were performed by diluting BRV plasmid standards to 4.51 × 10^5^ and 4.51 × 10^6^ copies/μL. Intra- and inter-batch CVs were less than 1.33% (Table 1), which was 0.48 times higher than that of qPCR. This indicated that the established BEV, BCoV, and BRV qPCR and ddPCR assays all had good reproducibility and stability, with the ddPCR assays for BEV and BRV being the most stable.

**Table 1:** Droplet digital PCR (ddPCR) repeatability.

	BEV				BCoV				BRV			
	Plasmid concentration (copies/μL)	Copy number (copies/μL)	SD	CV/%	Plasmid concentration (copies/μL)	Copy number (copies/μL)	SD	CV/%	Plasmid concentration (copies/μL)	Copy number (copies/μL)	SD	CV/%
Repeat within batch	1.52 × 10^6^	345.5	2.12	0.61	3.95 × 10^6^	8,150	212.13	2.6	4.51 × 10^6^	6,570	87.18	1.33
	1.52 × 10^5^	37.5	0.42	1.13	3.95 × 10^5^	3,340	14.14	0.42	4.51 × 10^5^	502.33	3.21	0.64
Inter-batch duplication	1.52 × 10^6^	343	3.53	1.03	3.95 × 10^6^	7,890	367.7	4.66	4.51 × 10^6^	6564.44	78.48	1.2
	1.52 × 10^5^	37.85	0.49	1.3	3.95 × 10^5^	3,465	176.78	5.1	4.51 × 10^5^	499.89	4.53	0.91

BEV, bovine enterovirus; BCoV, bovine coronavirus; BRV, bovine rotavirus; CT, cycle threshold; SD, standard deviation; CV, coefficient of variation.

### 3.5. Establishment of the BCoV and BRV dual ddPCR reaction system

**Figure 4:**
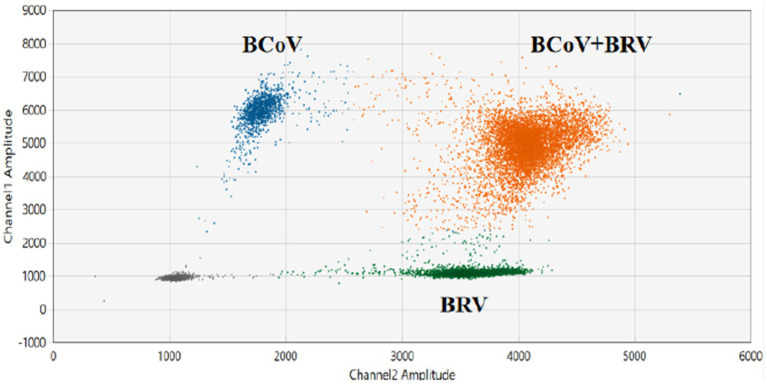
Establishment of the BCoV and BRV dual ddPCR detection systems. 2D plot of BCoV and BRV dual ddPCR results; blue: BCoV-positive droplets; green: BRV-positive droplets; orange: BCoV and BRV double-positive droplets; gray: negative. The horizontal and vertical coordinates are the fluorescence amplitudes of HEX and FAM, respectively. Channel 1 amplitude: FAM, respectively; channel 2 amplitude: HEX, respectively.

The results of the dual ddPCR reactions for BCoV and BRV detection (Figure 4; Supplementary Figures S3A,B) showed that the blue droplets indicate a positive for BCoV, the green droplets indicate a positive for BRV, and the orange droplets indicate a double positive for BCoV and BRV. The results showed that the dual ddPCR reaction system for BCoV and BRV established in this experiment could detect BCoV, BRV, or BCoV and BRV simultaneously.

### 3.6. Establishment and optimization of multiplex ddPCR assays for BEV, BCoV, and BRV

**Figure 5:**
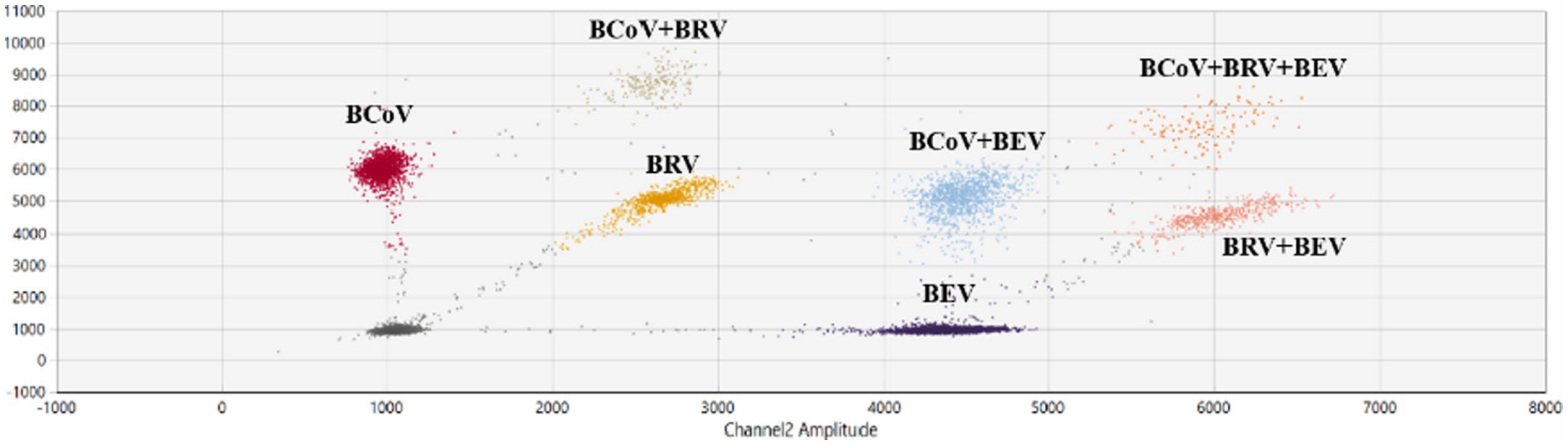
Establishment and optimization results of multiplex ddPCR assays for BEV, BCoV, and BRV. Multiplex ddPCR results for probe concentrations: BCoV FAM 200 nM, BRV FAM 100 nM, HEX 100 nM, and BEV HEX 200 nM; at an annealing temperature of 58°C and a primer concentration of 500 nM. Red: BCoV-positive droplets; yellow: BRV-positive droplets; purple: BEV-positive droplets; orange red: BRV and BEV double-positive droplets; brown: BCoV and BRV double-positive droplets; blue: BCoV and BEV double-positive droplets; orange: BEV, BCoV, and BRV triple-positive droplets; gray: negative. The horizontal and vertical coordinates are the fluorescence amplitudes of HEX and FAM, respectively. Channel 1 amplitude: FAM, respectively; channel 2 amplitude: HEX, respectively.

The reaction annealing temperature was set to 58°C, the primer concentration was 500 nM, and different probe concentrations were performed in the assay (Supplementary Table S3). The results showed that there were single-positive droplets, double-positive droplets, and triple-positive droplets, and the droplets appeared at the probe concentrations of BCoV FAM 200 nM, BRV FAM 100 nM, HEX 100 nM, and BEV HEX 200 nM. Droplet boundaries were obvious and concentrated (Figure 5), so this probe concentration, annealing temperature, and primer concentration were selected as the best reaction conditions. Other probe concentration results are plotted in Supplementary Figures S4A–C.

### 3.7. The detection of clinical samples

**Figure 6:**
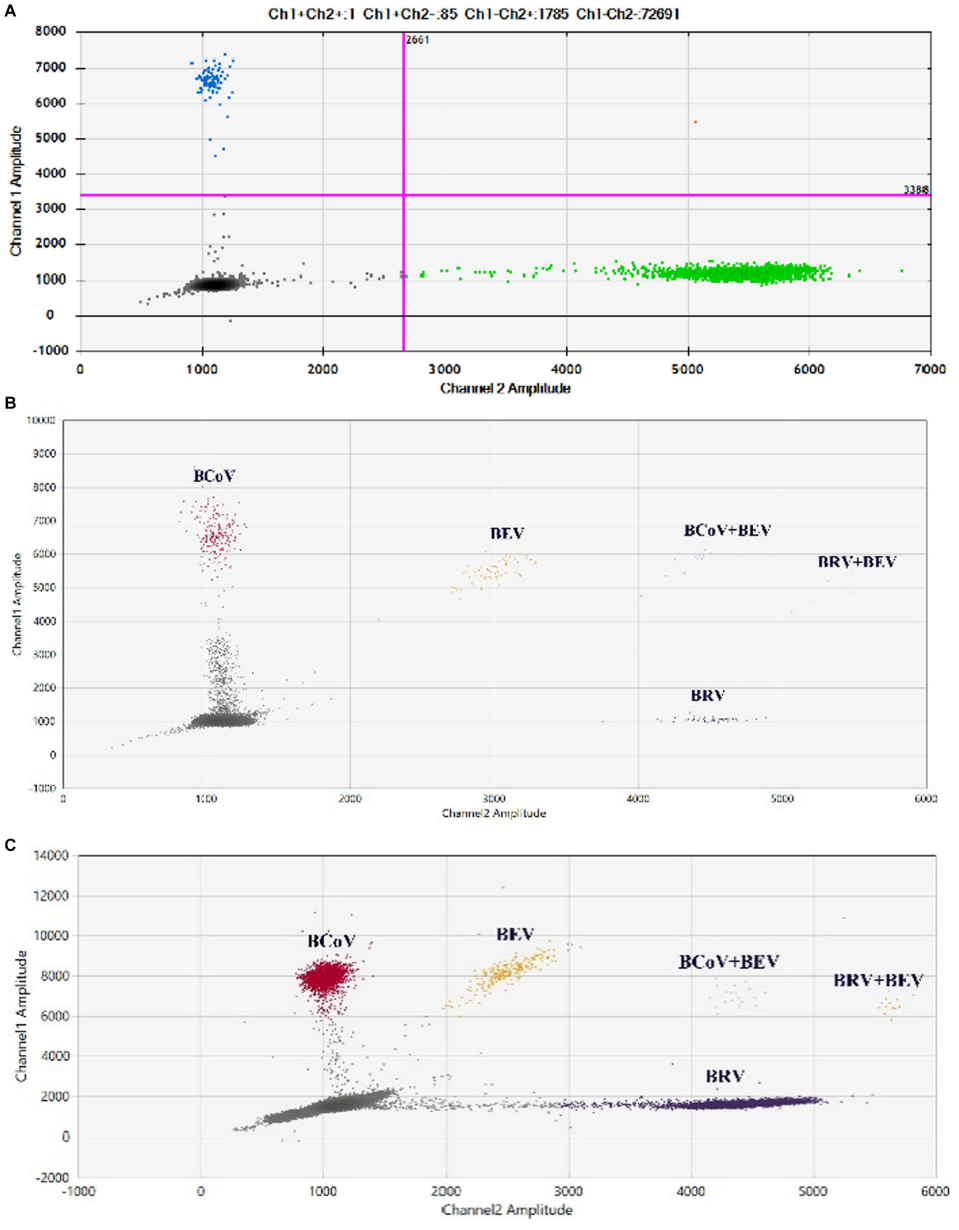
Results of clinical samples tested by duplex and multiplex ddPCR. **(A)** Results of co-infection in clinical samples tested by duplex ddPCR for BCoV and BRV; blue: BRV-positive droplets; green: BCoV-positive droplets; red: BRV and BCoV double-positive droplets. **(B,C)** Results of clinical tests by multiplex ddPCR for BCoV, BEV, and BRV. **(B)** Test results of 68 samples collected in Bole, Xinjiang, China. **(C)** Test results of 93 samples collected in Ürümqi County, Xinjiang, China. Red: BCoV-positive droplets; yellow: BRV-positive droplets; purple: BEV-positive droplets; orange: BRV and BEV double-positive droplets; blue: BCoV and BEV double-positive droplets; gray: negative. The horizontal and vertical coordinates are the fluorescence amplitudes of HEX and FAM, respectively. Channel 1 amplitude: FAM, respectively; channel 2 amplitude: HEX, respectively.

Eighty two anal swabs collected from diarrheal calves were tested using both the established qPCR and duplex ddPCR methods. The results showed that the positivity rates of qPCR for BCoV and BRV were 10.98% and 12.2%, respectively, with a 3.66% rate of co-infection. The positivity rates of BCoV and BRV by duplex ddPCR were 18.29% and 14.63%, respectively, with a 6.1% rate of co-infection (Figure 6A). The sensitivity was 1.66 times higher than that of qPCR for mixed infection detection (Table 2; Supplementary Table S8). Compared with the two methods, the dual ddPCR assay had better detection efficiency, further indicating that the ddPCR assay was more sensitive and reliable than qPCR.

**Table 2:** Comparison results of qPCR and double ddPCR clinical samples.

	qPCR positive rate	Double ddPCR positive rate	Sensitivity (times)
BCoV	10.98% (9/82)	18.29% (15/82)	1.66
BRV	12.2% (10/82)	14.63% (12/82)	1.2
BCoV and BRV	3.66% (3/82)	6.1% (5/82)	1.66

BCoV, bovine coronavirus; BRV, bovine rotavirus.

The established qPCR and multiplex ddPCR methods were used to test 68 anal swabs collected from diarrheal calves. The results showed that the positivity rates of qPCR for BCoV, BRV, and BEV were 14.49%, 1.45%, and 5.80%, respectively, with a 1.45% rate of co-infection for BCoV and BEV. The positivity rates of multiplex ddPCR for BCoV, BRV, and BEV were 14.49%, 2.9%, and 8.70%, respectively, with a co-infection rate of 2.9% for BCoV and BEV and 1.45% for BRV and BEV (Figure 6B). Multiplex ddPCR was twice as sensitive as qPCR for BRV detection, 1.5 times as sensitive as qPCR in detecting BEV, and twice the sensitive as qPCR in detecting co-infections with BCoV and BEV; qPCR did not detect samples with mixed BRV and BEV infections, while multiplex ddPCR detected one case (Table 3; Supplementary Table S9).

**Table 3:** Comparison results of qPCR and multiplex ddPCR clinical samples.

	qPCR positive rate	Multiplex ddPCR positive rate	Sensitivity (times)
BCoV	14.49% (10/68)	14.49% (10/68)	1
BRV	1.45% (1/68)	2.9% (2/68)	2
BEV	5.8% (4/68)	8.7% (6/68)	1.5
BCoV and BEV	1.45% (1/68)	2.9% (2/68)	2
BRV and BEV	—	1.45% (1/68)	—

BEV, bovine enterovirus; BCoV, bovine coronavirus; BRV, bovine rotavirus.

Ninety three anal swabs collected from Ürümqi County were tested. The results showed that the positive rates of qPCR for BCoV, BRV, and BEV were 5.38%, 1.08%, and 18.28%, respectively; among them, the co-infection rate of BCoV and BEV was 1.08%. The positive rates of multiplex ddPCR for BCoV, BRV, and BEV were 5.38%, 2.15%, and 20.45%, respectively; the co-infection rate for BCoV and BEV was 1.08% and for BRV and BEV was 1.08% (Figure 6C). Multiplex ddPCR was twice as sensitive as qPCR in detecting BRV and 1.12 times more sensitive than qPCR in detecting BEV. qPCR did not detect samples with mixed BRV and BEV infections, while multiplex ddPCR detected one case (Table 4; Supplementary Table S10).

**Table 4:** Comparison results of qPCR and multiplex ddPCR.

	qPCR positive rate	Multiplex ddPCR positive rate	Sensitivity (times)
BCoV	5.38% (5/93)	5.38% (5/93)	1
BRV	1.08% (1/93)	2.15% (2/93)	2
BEV	18.28% (17/93)	20.43% (19/93)	1.12
BCoV and BEV	1.08% (1/93)	1.08% (1/93)	1
BRV and BEV	—	1.08% (1/93)	—

BEV, bovine enterovirus; BCoV, bovine coronavirus; BRV, bovine rotavirus.

## 4. Discussion

Calf diarrhea is a common and frequent disease in cattle rearing and can be divided into two main categories based on the causes: infectious diseases, such as viral, bacterial, and parasitic infections, and non-infectious diseases, related to, for instance, environmental discomfort, nutrition, or management (20, 21). Among these causes, diarrhea caused by viruses is the most dangerous and difficult to prevent. The most common pathogens causing viral diarrhea in China are BEV, BCoV, BRV, and bovine viral diarrhea virus (BVDV) (22, 23). These infections cause diarrhea and a variety of complications in the body, leading to calf death in severe cases (24, 25). BEV, BCoV, and BRV infections have a high degree of clinical similarity, so the diagnosis of viral diarrhea epidemics cannot be confirmed by clinical diagnosis alone (26), requiring laboratory tests to determine the etiology. The most common method for laboratory testing of BEV, BCV, and BEV is PCR, but PCR has low sensitivity and poor specificity, which is not conducive to early detection and treatment of the disease. Therefore, there is an urgent need of a highly sensitive, specific, fast, and effective method for detection.

One of the most common methods in molecular biology diagnosis is the PCR assay (27). Ji et al. (28) established a quadruple one-step RT-PCR assay for BVDV, BEV, BRV, and BCoV, which is inexpensive and efficient, but the test result analysis requires the use of gel, which is prone to contamination with false positives, has low sensitivity, and cannot be quantified. Liu et al. (29) established a quadruple real-time fluorescence qPCR assay for BAstV, BVDV-1, BCoV, and BRV, which is fast, efficient, and can be quantified, but it relies on Ct values, which are easily affected by the amplification efficiency, and the construction of the standard curve needs to be calculated based on the Ct value, which leads to a decrease in accuracy (26, 30–32). The effective range of Ct values is between 15–35. When the template concentration is too high, the Ct value is less than 15, making the amplification not reach the fluorescence threshold within the baseline period, and when the template concentration is too low, the Ct value is greater than 35, which is considered meaningless. By using the established qPCR assay with gradient optimization of annealing temperature and primer concentration, the ddPCR assay for BEV, BCoV, and BRV was constructed using FAM and HEX fluorescent groups involved in the reaction. Due to the absence of a BRV strain, the constructed standard plasmids were used to the specificity assay. The results showed that only the positive plasmids can produce fluorescent signals, indicating good specificity. in terms of reproducibility, the intra- and inter-batch CVs for BEV, BCoV, and BRV qPCR assays were less than 3%; the CVs for BEV ddPCR were less than 1.3%, 0.5 times that of qPCR; the CVs for BCoV ddPCR were less than 5.1%, 2.13 times that of qPCR; and the CV of BRV was less than 1.33%, 2.13 times that of qPCR. This demonstrated that the established qPCR and ddPCR assays for BEV, BCoV, and BRV had good reproducibility and stability, with the ddPCR assays for BEV and BRV being the most stable. In terms of sensitivity, the lower limits of the established qPCR assays were all 1,000 copies/μL, while the lowest concentrations in the established ddPCR assays for BEV, BCoV, and BRV were 2.7 copies/μL, 1 copy/μL and 2.4 copies/μL, respectively; these values are 370.37, 1,000, and 416.67 times higher than those of the corresponding qPCR assays. The test detected a lower viral load and reduced false negatives. ddPCR also showed a negative fluorescent signal when the plasmid concentration was too high, indicating that ddPCR has an upper limit of detection. A review of related data revealed (33) that the principles of ddPCR are based on the Poisson distribution, which means that plasmid concentrations exceeding the upper limit of detection cannot be corrected by the system. This results in the inability to achieve accurate quantification of ddPCR, so a negative droplet must be present in the ddPCR reaction system. The concentrations in our plasmid tests were all between 1 × 10^6^–1 × 10^−1^ copies/μL, which did not exceed the upper limit of detection.

A study by Chang et al. (34) revealed that co-infection with BRV and BCoV is more serious, so a method that can detect BCoV and BRV simultaneously was imperative. A dual ddPCR assay for BCoV and BRV was established based on the previous ddPCR. The interactions between primers and probes in dual ddPCR are more complex compared to the single ddPCR detection system, and the quality of primer and probe design is the key to the successful implementation of duplex ddPCR. Referring to the SARS-CoV-2 multiplex ddPCR assay established by Nyaruaba et al. (18), the ratio of probes FAM and HEX in the duplex ddPCR was 1:1. The test results showed the presence of double positive droplets, indicating the successful establishment of the method. The established qPCR method and duplex ddPCR method were both used to test the batch of 82 calf diarrhea samples, and qPCR showed that the positive detection rate was 10.98% for BCoV and 12.2% for BRV. Duplex ddPCR showed that the positivity rate was 18.29% for BCoV, 7.31% higher than qPCR, and 14.63% for BRV, 2.43% higher than qPCR, with a 6.1% rate of co-infection, 2.44% higher than qPCR. This showed that the duplex ddPCR assay was not only able to detect positive samples, but it was also more sensitive than qPCR.

The multiplex ddPCR assay for BEV, BCoV, and BRV was further developed and optimized to achieve an annealing temperature of 58°C, using a primer concentration of 500 nM and probe concentrations of 200 nM for BCoV FAM, 100 nM for BRV FAM, 100 nM for HEX, and 200 nM for BEV HEX. The remaining 68 samples were tested using qPCR and the established multiplex ddPCR. qPCR showed positivity rates of 14.49% for BCoV, 1.45% for BRV, and 5.80% for BEV, with a 1.45% rate of co-infection between BCoV and BEV. Multiplex ddPCR showed positive rates of 14.49% for BCoV, 1.45% for BRV, and 8.70% for BEV, with a 2.9% rate of co-infection for BCoV and BEV and 1.45% for BRV and BEV; qPCR did not detect samples with BRV and BEV co-infection, while multiplex ddPCR showed a positivity rate of 1.45%. In another field, qPCR assays showed positive rates of 5.38%, 1.08%, and 18.28% for BCoV, BRV, and BEV, respectively; with a co-infection rate of 1.08% for BCoV and BEV. Multiplex ddPCR showed positive rates of 5.38%, 2.15%, and 20.45% for BCoV, BRV, and BEV, respectively; the co-infection rate was 1.08% for BCoV and 1.08% for BRV and BEV, where qPCR did not detect samples with mixed infection of BRV and BEV, while multiplex ddPCR detected one case.

## 5. Conclusion

This study shows how to develop a multiplex ddPCR test by optimizing the screening conditions for a single ddPCR system. This was determined to be a specific, sensitive and convenient method for simultaneous detection of BEV, BCoV, and BRV. It was able to reduce the number of false positive and false negative results during testing and contributed to the development of better diagnostic methods.

## Data availability statement

The original contributions presented in the study are included in the article/Supplementary material, further inquiries can be directed to the corresponding author.

## Ethics statement

Samples for this study were collected with the consent of the animal owners, and no live animals were handled in the study, so no ethical approval was required for this study.

## Author contributions

QF, DL, and CZ contributed to the conception and design of the study. DL and YX organized the database. YY and CZ made the sampling and processing of the studied materials. SM, XL, and ZL performed the statistical analysis. JC wrote the first draft of the manuscript. HS is the main tutor of the study and he reviewed the results obtained and the manuscripts. All authors contributed to the article and approved the submitted version.

## Funding

This study was supported by the Autonomous Region Outstanding Youth Fund Project (grant number: 2022D01E15); Autonomous Region Major Science and Technology Project (grant number: 2020A01001-2); National Natural Science Foundation of China (grant number: 32260881); National Student Innovation Project (grant number: 202110758005), Autonomous Region Postgraduate Innovation Project (grant number: XJ2022G131).

## Conflict of interest

DL was employed by Tecon Biology Co., Ltd.

The remaining authors declare that the research was conducted in the absence of any commercial or financial relationships that could be construed as a potential conflict of interest.

## Publisher’s note

All claims expressed in this article are solely those of the authors and do not necessarily represent those of their affiliated organizations, or those of the publisher, the editors and the reviewers. Any product that may be evaluated in this article, or claim that may be made by its manufacturer, is not guaranteed or endorsed by the publisher.
