# Supplementary material for: Establishment and application of multiplex droplet digital polymerase chain reaction assay for bovine enterovirus, bovine coronavirus, and bovine rotavirus
Source: Front Vet Sci. 2023 Sep 12;10:1157900. doi: 10.3389/fvets.2023.1157900 (PMC10523346; doi:10.3389/fvets.2023.1157900)
Supplement: Supplementary file 1 [file Data_Sheet_1.docx]

Supplementary Material

Establishment and Application of Multiplex Droplet Digital PCR Assay for bovine enterovirus, bovine coronavirus, and bovine rotavirus

Junzhen Chen^†^, Dan Li^†^, Yafang Xu, Zeyu Li, xinyi Liu, Yuanyuan Yuan, Chengyuan Zhang, Qiang Fu, Huijun Shi*

*** Correspondence:** Huijun Shi: [shihuijunmm@163.com](mailto:shihuijunmm@163.com)

# Figure 1.

| A | B | C |
| --- | --- | --- |
| 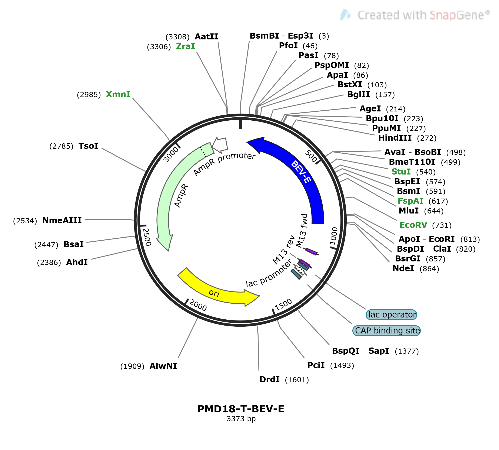 | 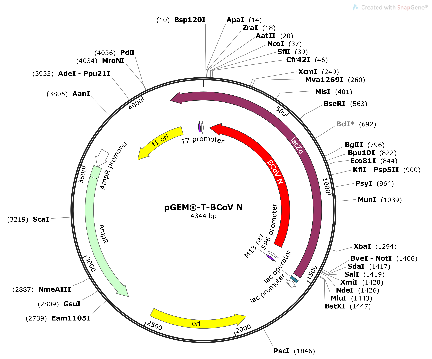 | 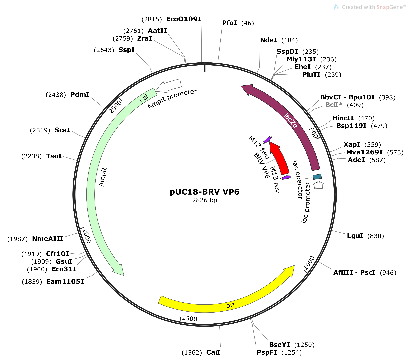 |

Figure 1. Recombinant plasmid mapping. A: pMD18-T-BEV-E; B: pGEM-T-BCoV-N; C: pUC18-BRV-VP6.

# Primer and probe sequences

Table 1 PCR reaction system

| Reagent name | consumption（μL） |
| --- | --- |
| 2 × Taq PCR Mix | 12.5 |
| Primer-F | 1 |
| Primer-R | 1 |
| Template | 2 |
| RNase-Free ddH2O | Up to 20 |

F: Forward primers; R: Reverse primers.

# Primer and probe sequences

Table 2 Primer and probe sequences

| Primer/Probe | sequence（5’→3’） | Product size（bp） |
| --- | --- | --- |
| BEV-F | CGCAATGGTCAAGCACTTCT | 80 |
| BEV-R | GGCCATACTAGGCTTCTCGT |  |
| BEV-Probe | FAM-CGCCCAGCGTCGTTACCCGC-BHQ1 |  |
| BCoV-F | GATCTACTTCACGCGCATCC | 159 |
| BCoV-R | GTGGCTTAGTGGCATCCTTG |  |
| BCoV-Probe | HEX-TGGCTCTACTGCGCGATCCTGCA-BHQ1 |  |
| BRV- F | CAATGTGGCTGAATGCAGGA | 127 |
| BRV- R | GCGGTAGTCAACACTCTTCG |  |
| BRV-Probe-HEX | HEX-TGCGCTATCAACGCACCAGCT-BHQ1 |  |
| BRV-Probe-FAM | FAM-TGCGCTATCAACGCACCAGCT-BHQ1 |  |

BEV: Bovine enterovirus; BCoV: Bovine coronavirus; BRV: Bovine rotavirus; F: Forward primers; R: Reverse primers.

# Optimization of probe concentrations for BEV, BCoV and BRV Multiplex ddPCR

Table 3 Sequence and concentration of probes

| Probe | Sequence | | Concentration(nM) | | | |
| --- | --- | --- | --- | --- | --- | --- |
|  |  |  | A | B | C | D |
| BEV-Probe | | FAM-CGCCCAGCGTCGTTACCCGC-BHQ1 | 200 | 300 | 300 | 200 |
| BRV-Probe-FAM | | FAM-TGCGCTATCAACGCACCAGCT-BHQ1 | 100 | 100 | 100 | 200 |
| BRV-Probe-HEX | | HEX-TGCGCTATCAACGCACCAGCT-BHQ1 | 100 | 200 | 100 | 200 |
| BCoV-Probe | | HEX-TGGCTCTACTGCGCGATCCTGCA-BHQ1 | 200 | 300 | 300 | 200 |

BEV: Bovine enterovirus; BCoV: Bovine coronavirus; BRV: Bovine rotavirus.

# Results of qPCR primer concentration optimization

Table 4 Primer concentration optimization for BEV, BCoV and BRV

| Primer concentration（nM） | BEV（Ct value） | BCoV（Ct value） | BRV（Ct value） |
| --- | --- | --- | --- |
| 200 | 18.84 | 23.9 | 23.62 |
| 300 | 18.91 | 23.89 | 23.54 |
| 400 | 18.93 | 24.88 | 23.81 |
| 500 | 18.95 | 23.96 | 24.02 |
| 600 | 19.92 | 23.95 | 23.68 |
| 700 | 19.91 | 23.9 | 23.75 |
| 800 | 19.96 | 23.96 | 23.6 |
| 900 | 19.02 | 23.87 | 23.77 |

BEV: Bovine enterovirus; BCoV: Bovine coronavirus; BRV: Bovine rotavirus; CT: Cycle Threshold.

# Results of qPCR Probe concentration optimization

Table 5 Probe concentration optimization for BEV, BCoV and BRV

| Probe concentration（nM） | BEV（Ct value） | BCoV（Ct value） | BRV（Ct value） |
| --- | --- | --- | --- |
| 150 | 18.92 | 23.94 | 24.03 |
| 200 | 18.97 | 23.96 | 23.72 |
| 250 | 18.95 | 23.97 | 23.48 |
| 300 | 18.96 | 24.93 | 23.3 |
| 350 | 18.98 | 24.93 | 23.25 |
| 400 | 18.83 | 24.91 | 23.21 |
| 450 | 18.92 | 24.94 | 23.14 |
| 500 | 18.96 | - | 23.05 |

BEV: Bovine enterovirus; BCoV: Bovine coronavirus; BRV: Bovine rotavirus; CT: Cycle Threshold.

# Sensitivity of qPCR for detecting BEV, BCoV and BRV

Table 6 Sensitivity of qPCR for detecting BEV, BCoV and BRV

| Number of copies（copies/μL） | BEV（Ct value） | BCoV（Ct value） | BRV（Ct value） |
| --- | --- | --- | --- |
| 1×10^9^ | 12.26 | 12.08 | 14.07 |
| 1×10^8^ | 15.99 | 15.47 | 17.3 |
| 1×10^7^ | 18.77 | 18.92 | 20.65 |
| 1×10^6^ | 22.16 | 22.68 | 24.15 |
| 1×10^5^ | 25.61 | 26.27 | 27.37 |
| 1×10^4^ | 28.89 | 29.83 | 30.84 |
| 1×10^3^ | 32.41 | 33.05 | 33.92 |
| 1×10^2^ | 35.66 | 36.09 | 37.8 |

BEV: Bovine enterovirus; BCoV: Bovine coronavirus; BRV: Bovine rotavirus; CT: Cycle Threshold.

# Figure 2.

| (A) | 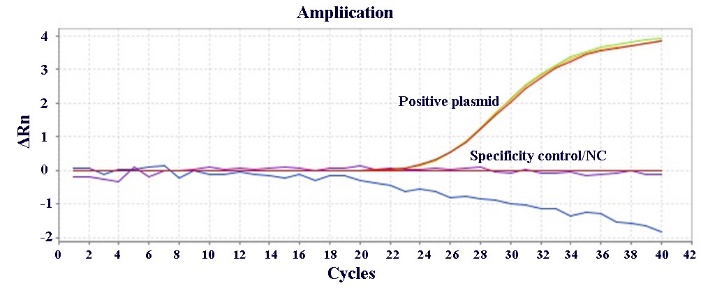 |
| --- | --- |
| (B) | 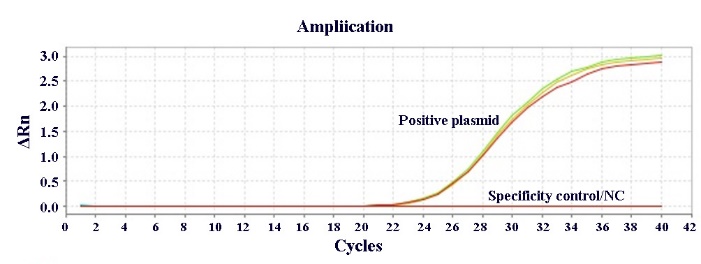 |
| (C) | 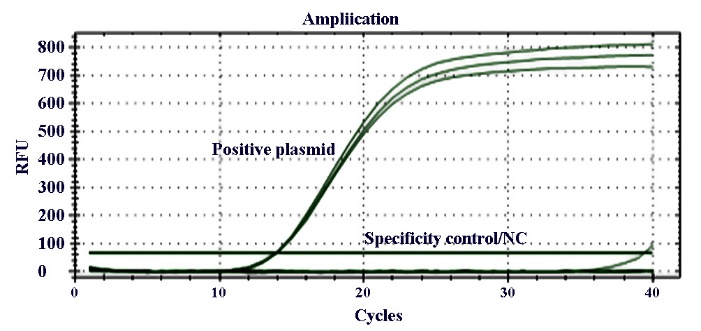 |

Figure 2. Specificity detection results of qPCR assays. Specificity detection of BEV, BCoV, BRV, and H_2_O using established qPCR assays. A: Specificity detection of BEV using qPCR; B: Specificity detection of BCoV using qPCR; C: Specificity detection of BRV using qPCR. Rn: Intensitas fluoresensi. RFU: Relative fluorescence units.

# qPCR repeatability

Table 7 qPCR repeatability

|  | BEV | | | | BCoV | | | | BRV | | | |
| --- | --- | --- | --- | --- | --- | --- | --- | --- | --- | --- | --- | --- |
|  | Plasmid concentration（copies/μL） | Mean Ct | SD | CV/% | Plasmid concentration（copies/μL） | Mean Ct | SD | CV/% | Plasmid concentration（copies/μL） | Mean Ct | SD | CV/% |
| Repeat within batch | 1 × 10^7^ | 19.98 | 0.02 | 0.09 | 1 × 10^7^ | 19.94 | 0.03 | 0.15 | 1 × 10^7^ | 20.58 | 0.1 | 0.49 |
|  | 1 × 10^6^ | 23.88 | 0.12 | 0.5 | 1 × 10^6^ | 22.96 | 0.02 | 0.08 | 1 × 10^6^ | 24.16 | 0.12 | 0.49 |
|  | 1 × 10^5^ | 26.87 | 0.09 | 0.35 | 1 × 10^5^ | 25.92 | 0.04 | 0.16 | 1 × 10^5^ | 27.69 | 0.08 | 0.29 |
| Inter-batch duplication | 1 × 10^7^ | 20.59 | 0.53 | 2.6 | 1 × 10^7^ | 19.56 | 0.47 | 2.4 | 1 × 10^7^ | 20.12 | 0.57 | 2.82 |
|  | 1 × 10^6^ | 23.88 | 0.01 | 0.03 | 1 × 10^6^ | 22.92 | 0.05 | 0.2 | 1 × 10^6^ | 23.84 | 0.52 | 2.2 |
|  | 1 × 10^5^ | 26.91 | 0.06 | 0.24 | 1 × 10^5^ | 25.92 | 0.04 | 0.15 | 1 × 10^5^ | 27.26 | 0.55 | 2 |

BEV: Bovine enterovirus; BCoV: Bovine coronavirus; BRV: Bovine rotavirus; CT: Cycle Threshold; SD: Standard Deviation; CV: Coefficient of variation.

# Figure 3.

| (A) | 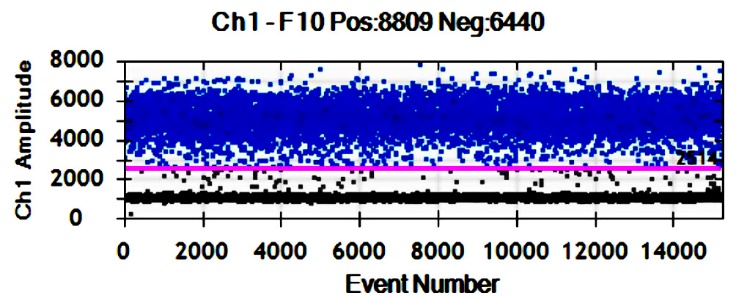 |
| --- | --- |
| (B) | 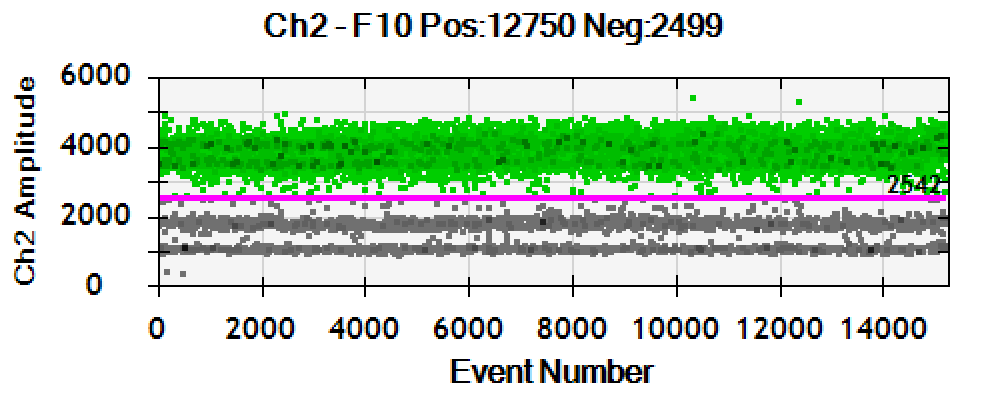 |

Figure 3. Establishment of BCoV and BRV dual ddPCR detection system. A: 1D plot of BCOV FAM fluorescence channel; B: 1D plot of BRV HEX fluorescence channel; Ch 1 Amplitude: FAM respectively; Ch 2 Amplitude: HEX respectively.

# Figure 4.

| (A) | 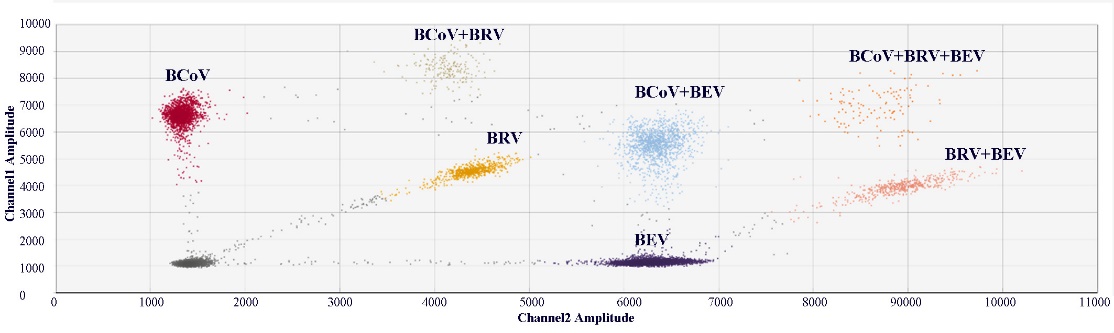 |
| --- | --- |
| (B) | 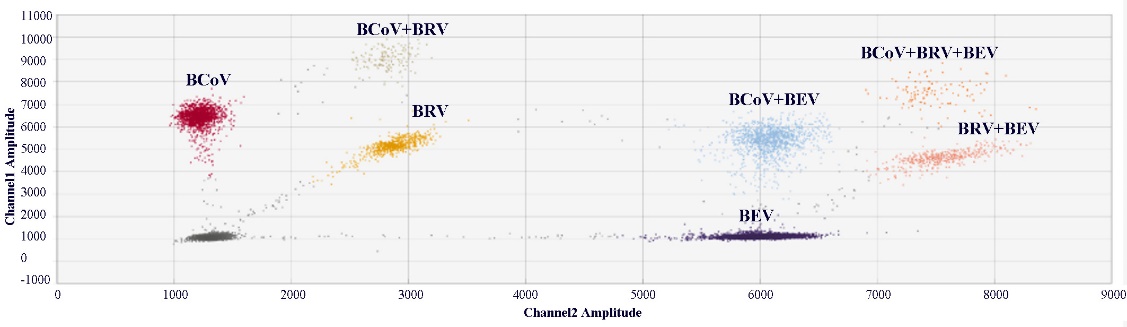 |
| (C) | 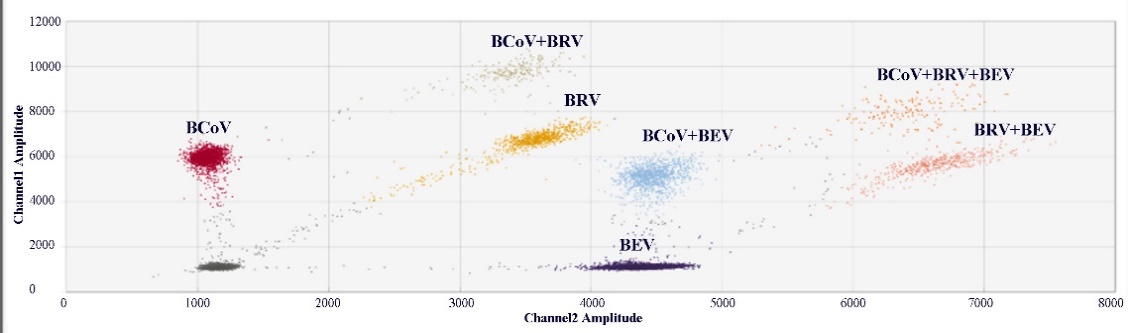 |

Figure 4. Establishment and optimization results of multiplex ddPCR assays for BEV, BCoV, and BRV. Multiplex ddPCR results for different probe concentrations, (A) probe concentrations in additional Table S2 for group B; (B) probe concentrations in additional Table S2 for group C; (C) probe concentrations in additional Table S2 for group D; At annealing temperature of 58 °C and primer concentration of 500 nM. Red: BCoV-positive droplets; Yellow: BRV-positive droplets; Purple: BEV-positive droplets; Orange red: BRV and BEV double-positive droplets; Brown: BCoV and BRV double-positive droplets; Blue: BCoV and BEV double-positive droplets; Orange: BEV, BCoV, and BRV triple-positive droplets; Gray: negative. The horizontal and vertical coordinates are the fluorescence amplitudes of FAM and HEX, respectively. Channel 1 Amplitude: FAM respectively; Channel 2 Amplitude: HEX respectively.

# The detection of clinical samples

Table 8 82 samples tested positive with serial number

|  | qPCR positive number | ddPCR positive number |
| --- | --- | --- |
| BCoV | H2、H7、H18、H22、H38、H46、H55、H67、H77 | H2、H7、H12、H18、H22、H38、H39、H46、H48、H55、H58、H67、H69、H77、H80 |
| BRV | H3、H7、H15、H19、H20、H22、H26、H35、H41、H67 | H3、H7、H15、H19、H20、H22、H26、H35、H39、H41、H46、H67 |
| BCoV and BRV | H7、H22、H67 | H7、H22、H39、H46、H67 |

BCoV: Bovine coronavirus; BRV: Bovine rotavirus.

# The detection of clinical samples

Table 9 68 samples tested positive with serial number

|  | qPCR positive number | ddPCR positive number |
| --- | --- | --- |
| BCoV | A3、A9、A14、A19、A26、A36、A38、A55、A57、A67 | A3、A9、A14、A19、A26、A36、A38、A55、A57、A67 |
| BRV | A6 | A6、A20 |
| BEV | A9、A25、A34、A50 | A9、A20、A25、A34、A50、A67 |
| BCoV and BEV | A9 | A9、A67 |
| BRV and BEV |  | A20 |

BEV: Bovine enterovirus; BCoV: Bovine coronavirus; BRV: Bovine rotavirus.

# The detection of clinical samples

Table 10 93 samples tested positive with serial number

|  | qPCR positive number | ddPCR positive number |
| --- | --- | --- |
| BCoV | N26、N42、N49、N81、N82 | N26、N42、N49、N81、N82 |
| BRV | N42 | N42、N90 |
| BEV | N8、N9、N27、N29、N32、N36、N38、N45、N47、N48、N60、N83、N84、N85、N86、N87、N88 | N6、N8、N9、N27、N29、N32、N36、N38、N45、N47、N48、N60、N83、N84、N85、N86、N87、N88、N90 |
| BCoV and BEV | N42 | N42 |
| BRV and BEV |  | N90 |

BEV: Bovine enterovirus; BCoV: Bovine coronavirus; BRV: Bovine rotavirus.

**
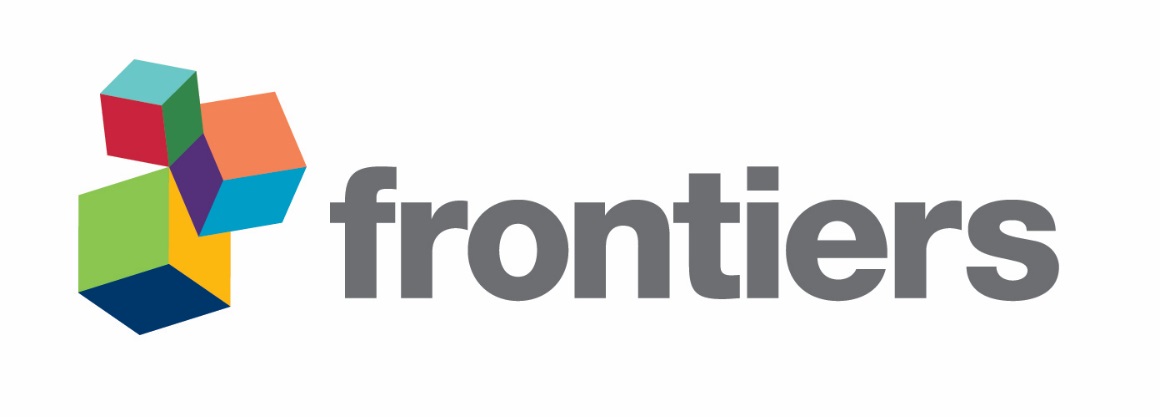
**
